# The Impact of Far-Red Light Supplementation on Hormonal Responses to Cold Acclimation in Barley

**DOI:** 10.3390/biom11030450

**Published:** 2021-03-17

**Authors:** Mohamed Ahres, Tamás Pálmai, Krisztián Gierczik, Petre Dobrev, Radomíra Vanková, Gábor Galiba

**Affiliations:** 1 Centre for Agricultural Research, Agricultural Institute, Eötvös Loránd Research Network, H-2462 Martonvásár, Hungary; mohamed.ahres@atk.hu (M.A.); palmai.tamas@atk.hu (T.P.); gierczik@gmail.com (K.G.); galiba.gabor@atk.hu (G.G.); 2 Department of Environmental Sustainability, Festetics Doctoral School, IES, Hungarian University of Agriculture and Life Sciences, H-8360 Keszthely, Hungary; 3 Institute of Experimental Botany of the Czech Academy of Sciences, 165 02 Prague, Czech Republic; dobrev@ueb.cas.cz

**Keywords:** phytohormones, frost-tolerance, low R/FR ratio, LED lighting, barley

## Abstract

Cold acclimation, the necessary prerequisite for promotion of freezing tolerance, is affected by both low temperature and enhanced far-red/red light (FR/R) ratio. The impact of FR supplementation to white light, created by artificial LED light sources, on the hormone levels, metabolism, and expression of the key hormone metabolism-related genes was determined in winter barley at moderate (15 °C) and low (5 °C) temperature. FR-enhanced freezing tolerance at 15 °C was associated with promotion of abscisic acid (ABA) levels, and accompanied by a moderate increase in indole-3-acetic acid (IAA) and *cis*-zeatin levels. The most prominent impact on the plants’ freezing tolerance was found after FR pre-treatment at 15 °C (for 10 days) followed by cold treatment at FR supplementation (7 days). The response of ABA was diminished in comparison with white light treatment, probably due to the elevation of stress tolerance during FR pre-treatment. Jasmonic acid (JA) and salicylic acid (SA) were transiently reduced. When the plants were exposed directly to a combination of cold (5 °C) and FR supplementation, ABA increase was higher than in white light, and was associated with enhanced elevation of JA and, in the longer term (after 7 days), with IAA and *cis*-zeatin increase, which indicates a stronger stress response and better acclimation. Cold hardening was more efficient when FR light was applied in the early developmental stage of the barley plants (three-leaf stage, 18 days), rather than in later stages (28-days). The dynamics of the phytohormone changes are well supported by the expression profiles of the key hormone metabolism-related genes. This series of treatments serves as evidence for the close relationship between plant hormones, light quality, and low temperature at the beginning of cold acclimation. Besides the timing of the FR treatments, plant age also represents a key factor during light spectrum-dependent cold acclimation.

## 1. Introduction

The ability of plant species to acclimate to cold and to develop frost tolerance is a genetically determined physiological trait. This adaptation process, the so-called “cold-hardening” takes a relatively long time for overwintering plants in the temperate climate zone. In autumn, environmental changes (e.g., temperature, light intensity, and spectra) are required for cereals to prepare physiologically for winter frost. In the regulation of plant interactions with the altering environment, phytohormones have a pivotal role in controlling the coordination of the acclimation processes [1,2,3,4].

Based on their response to stress conditions, phytohormones can be divided into two groups. The first group consists of the so called “positive growth regulators” and includes auxins, cytokinins (CKs), gibberellins (GA), and brassinosteroids. The second group encompasses stress hormones with abscisic acid (ABA), jasmonic acid (JA), salicylic acid (SA), ethylene, and strigolactones [5].

One of the most important hormones in the responses to abiotic stresses is ABA, which is usually referred to as “the main abiotic stress hormone”. It has a pivotal role in the response to drought, salinity, and also to cold stress [6]. To avoid cold injury, the elevation of ABA content can stabilize plant water homeostasis in the early stages of the cold stress response and stimulate the production of protective compounds [7]. For example, in einkorn wheat (*Triticum monococcum ssp. monococcum* (L.)) the up-regulation of ABA was detected in crowns and leaves on the first day at 4 °C. This elevation promoted improvement of the plant water status via stomata closure, and promoted defense by stimulation of the expression of late embryogenesis abundant proteins (LEA proteins) [8]. In higher plants the starting point of the ABA biosynthesis pathway is the epoxidation of zeaxanthin and antheraxanthin to violaxanthin, which is catalyzed by a zeaxanthin epoxidase (ZEP) [9]. After the epoxidation, all-*trans*-violaxanthin is converted to 9-*cis*-violaxanthin or 9-*cis*-neoxanthin [10]. The next, rate-limiting step is the oxidative cleavage of 9-*cis*-violaxanthin and 9-*cis*-neoxanthin to xanthoxin catalyzed by 9-*cis*-epoxycarotenoid dioxygenase (NCED) [11,12,13]. The NCED genes seem to be very important in the cold adaptation process. In fact, in cold-treated wheat varieties the transcript level of *NCED1* was down-regulated in leaves in the freezing-sensitive variety, whereas it was highly elevated in the freezing-tolerant genotype [14]. After oxidative cleavage, xanthoxin is transported from the plastids to the cytosol and converted to abscisic aldehyde by short-chain dehydrogenase (SDR) [15,16]. In the final step, abscisic aldehyde is oxidized to ABA by aldehyde oxidase (AO) [17].

SA has been reported to play an important role in both biotic and abiotic stress responses in a wide range of plant species. In cereals, numerous studies have shown that SA treatment has a positive effect on low temperature tolerance [18]. In barley, exogenous SA application improved cold tolerance by decreasing lipid peroxidation, as well as ice nucleation, in both cold-tolerant and -sensitive varieties. SA also influenced the activities of apoplastic antioxidant enzymes [19,20]. Pre-treatment with SA at normal growth temperature induced similar defense mechanisms in maize as cold, namely increased antioxidant activity [21]. In addition, SA can also protect plants from oxidative stress by balancing their redox state [22,23]. The main SA biosynthesis pathway includes conversion of phenylalanine by phenylalanine ammonia lyase (PAL), the parallel pathway is catalysed by isochorismate synthase (ICS) [24,25]. PAL was found to be a stress-inducible enzyme up-regulated by UV irradiation, high light intensity, or low temperature [26]. In cucumber, stimulation of *PAL* expression correlated with reduced photo- and oxidative damages during cold stress [27]. In cereals, the results seem to be ambiguous. One study reported up-regulation of *PAL* in winter wheat during the whole cold-hardening period (2 °C for 28 days) [28], whereas another study on winter wheat showed a decrease of PAL activity during cold treatment [29].

JA is involved in the regulation of many physiological processes and has a crucial role in many biotic and abiotic stress responses [30,31]. Transcription of many JA biosynthetic genes, as well as JA content, was reported to increase during cold stress, e.g., in rice [32]. An elevated JA level was also observed upon prolonged cold exposure in winter and spring wheat varieties [5]. Similarly, JA content elevation was described upon drought stress [33]. The JA biosynthetic pathway starts with phospholipid release from plastid membrane lipids, followed by lipoxygenase (LOXs) and α-dioxygenase (α-DOXs) catalysis [34,35]. A potential role for LOX enzymes in cold acclimation was suggested in different plant species. However, freezing temperature treatment (−10 °C) led to great reduction of LOX activity in chickpea [36]. Prolonged heat or cold stress exposure led to significant changes in LOX-related volatile production in tomato [37].

Auxins are one of the most studied hormones in the field of plant growth and development, but very limited information is available about their role in cold acclimation. Most of the knowledge about the involvement of auxins in cold acclimation has been accumulated from *Arabidopsis thaliana* (L.) Heynh and rice studies [38,39,40]. The most biologically active auxin is indole-3-acetic acid (IAA) [41,42]. In general, prolonged cold stress positively influenced the accumulation of IAA, as well as the expression of many genes involved in auxin metabolism [5,32]. IAA can be synthesized by several pathways. One of the most important of these includes formation of indole-3-pyruvate (IPA) via the flavin monooxygenase (YUCCA) family, which is responsible for the decarboxylation of IPA into IAA [43].

The primary physiological functions of CKs are related to plant developmental processes (namely stimulation of cell division and enlargement), but they are also known to play a role in abiotic and biotic stress responses [44]. CKs were found to play a prominent role in cold protection [45] but their specific function in this area has not been fully elucidated yet. Usually, the amount of active CKs decreases immediately upon cold shock, which is related to the plant’s effort to relocate the energy from growth to defense [8,46]. There are four active CK forms in plants, namely *trans*-zeatin (tZ), *cis*-zeatin (cZ), isopentenyladenine (iP), and dihydrozeatin (DHZ) [47]. The physiologically most active CK in stimulation of cell division is tZ [48], whereas the less active cZ displays other CK functions, and can be linked to stress responses [47,49]. The key, rate-limiting CK biosynthesis genes are the isopentenyltransferases (IPTs). The main degradation enzymes are cytokinin oxidase/dehydrogenases (CKXs).

In spite of the fact that many studies have dealt with interactions between light-induced plant regulations and multiple hormonal pathways [50,51], little is known about hormonal changes due to the modifications of light quality during the cold acclimation processes. The most studied issue related to the effect of light spectrum composition is the “shade avoidance syndrome”. Plants can use light spectrum composition as information on the neighbouring vegetation [52]. Blue and red light are used for photosynthesis, thus their decreased ratio in proportion to far-red light (FR) in a dense canopy (given by absorption of red light (R) by the neighbouring plants and their reflection of FR) represents an important morphological signal. The elevated proportion of FR initiates the shade avoidance response via a number of molecular mechanisms, including up-regulation of auxins [50,53]. Decreased R/FR ratios associated with IAA elevation resulted in the upregulation of *cytokinin oxidase/dehydrogenase* 6 (*CKX6*) in young plant leaves, which led to CK down-regulation and suppression of leaf growth [54,55]. The decreased R/FR ratio also negatively influenced the accumulation of JA and SA, which was associated with enhanced plant vulnerability to pathogen attack [56,57].

However, apart from the shade effect, light-quality signals provide important seasonal information to plants as well. The effects of photoperiod on plant freezing tolerance and development are well established, whereas the possible influence of qualitative diurnal changes in the light environment has received less attention. The R/FR ratio is constant during daylight and independent of cloud cover but is much reduced during twilight, when solar elevation is less than 10^0^ [58]. Moving away from the equator, this phenomenon becomes more pronounced and characteristic of the temperate and boreal climates in the northern hemisphere. A reduced R/FR ratio during the twilight hours, especially in the evening, has been shown to control the cessation of internode elongation of aspen at the end of the active growing period, moreover twilight FR treatment advanced the leaf bud burst of silver birch [59,60].

Light quality, especially higher FR proportion was found to be an important factor in cold acclimation processes in *Arabidopsis thaliana* (L.). In our previous studies, we demonstrated that the decreased R/FR ratio positively influenced the freezing tolerance of wheat and barley cereals, which could be further enhanced by increasing the light intensity in the pre-hardening phase [61,62]. Light is perceived by plants via photoreceptors; R and FR by phytochromes [61,63,64,65]. The fact that the most abundant phytochrome, PhyB, functions as a thermosensor [66], indicates that the combination of high FR and low temperature represents for plants a unique environmental signal, differing, at least partially, from the shade avoidance response at optimal temperature. This conclusion is supported by specific transcription profiles, e.g., the synergistic effect of FR-stimulated PhyA and low temperature on the expression of the key cold-induced transcription factors, CBFs [67].

Both low temperature and light quality substantially influence the metabolism of many phytohormones, which subsequently affects the cold acclimation processes in plants. The aim of the present study was the elucidation of the mode of action of FR (applied by artificial LED light sources) at moderate and low temperature on freezing tolerance, via modulation of phytohormone pools. The hormone levels and their metabolism were compared in winter barley under moderate temperature +/- FR, during cold stress after FR pre-hardening, and upon direct application of combined FR and cold treatment. This study is part of a broader project on the evaluation of FR functions in cold hardening. A part of this project, focused on membrane lipids, was published recently [68].

## 2. Materials and Methods

### 2.1. Plant Materials and Growth Conditions

Winter barley with good resistance to cold temperature, *Hordeum vulgare spp. vulgare var.* Nure, was used in this study. After three days of germination, seedlings were planted in wooden boxes (30 cm × 25 cm × 10 cm) filled with soil. The boxes were placed into a PGV-36 growth chamber (Conviron PGV36; Controlled Environments Ltd.; Winnipeg, MB, Canada) equipped with a modular LED light ceiling. Plants were grown at constant 15 °C, with 12-h photoperiods for fourteen days. The light source was white light (W) provided by a continuous wide-spectrum LED (Philips Lumileds, LXZ25790-y) at 250 µmol m^−2^·s^−1^ intensity.

### 2.2. Light and Temperature Conditions during Experimental Treatments

The 18-d plants were separated into two areas. In one area white light at 250 µmol m^−2^·s^−1^ intensity was supplied, and the plants in this zone served as control, whereas in the other zone white light was supplemented by FR illumination, with a narrow 750 nm LED (Edison Edixeon, 2ER101FX00000001), so that the R/FR ratio was modified to 0.5 (Supplementary Figure S1, [68]). The control zones did not contain any FR illumination. The whole experiment consisted of three different variants.

**Figure 1:**
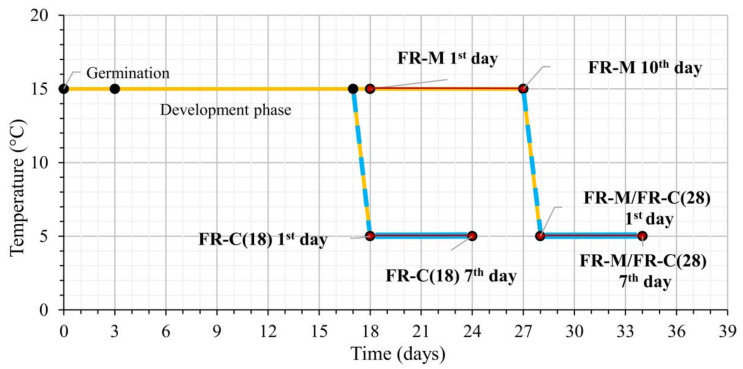
The experimental set-up describing the light and temperature treatments. Treatment 1 (FR-M): The plantlets were exposed to high far-red (FR) at the age of 18 days under constant 15 °C for 10 days. Treatment 2 (FR-M/FR-C(28)): The plantlets were subjected to high FR for 10 days at 15 °C, then the temperature was dropped to 5 °C for another seven days (at high FR). Treatment 3 (FR-C(18)): The plantlets were 18 days old when the temperature was dropped to 5 °C for seven days (at high FR). The red lines represent the FR treatments, while the blue lines indicate the low temperature 5 °C.

The treatment variants (see Figure 1):

1. The 18-day-old plants were exposed to FR at moderate temperature 15 °C for 10 days (FR-M). During FR exposition, the plants reached the four-leaf stage [69].
2. The 28-day-old (FR pre-hardened) plants from variant (1) were exposed to 5 °C for another 7 days (at high FR) [FR-M/FR-C(28)]. At the end of the stress treatment the plants were 34 days old.
3. The 18-day-old plants were exposed directly to a combination of cold 5 °C and high FR for 7 days [FR-C(18)].

Samples were collected during the first and the last days of each treatment during a roughly 2-h period between 6 to 8 a.m. (ZT6 to ZT8) daytime. Cold treatment was applied by gradually decreasing the temperature from 15 °C to 5 °C during the night, before the additional FR light was switched on in the morning.

### 2.3. Measurements of Electrolyte Leakage Levels in Leaf Samples

Freezing tests were performed according to [70]. The leaf samples were taken on the last day of each of the experimental variants. Two-mm-long leaf segments were sampled from each treatment. In the FR-M and FR-M/FR-C(28) treatments, twelve, and in FR-C(18) treatment, eight segments, were placed in 14-mL Falcon tubes (Thermo Fisher Scientific Inc., Wilmington, MA, USA). Leaf segments from four different plants were combined. Afterwards they were placed into a GP200-R4 liquid freezing system (Grant Instruments, Shepreth, England) in five biological repetitions per treatment and freezing point. To simulate cold acclimation (as under field conditions), the samples were kept at −2 °C for 18 h. Subsequently, the samples from FR-M and W control were frozen at −5, −7, and −9 °C, whereas the samples from FR-M/FR-C(28) and FR-C(18) (and the corresponding W controls) were frozen at −8, −10, and −12 °C for one hour. For the determination of electrolyte leakage, 8 mL MQ water was added to each tube. The samples were shaken gently for two hours before the measurements. Electrolyte leakage was determined by conductometer (Mikro KKT, Budapest, Hungary). For the data analysis, Multi-Sample Conductometer version 1.0 (Intron Software, Biological Research Centre, Szeged, Hungary (Copyright© L. Menczel, 2002)) was used.

### 2.4. Hormone Analysis

Leaf samples were purified and analyzed according to [71] and [72]. Samples were homogenized with a ball mill (MM301, Retsch) and extracted in cold (−20 °C) methanol/water/formic acid (15/4/1 *v/v/v*). The following labelled internal standards (10 pmol/sample) were added: ^13^C_6_-IAA, ^2^H_2_-OxIAA (Cambridge Isotope Laboratories); ^2^H_4_-SA (Sigma-Aldrich, St. Louis, MI, USA, ); ^2^H_3_-PA (phaseic acid), ^2^H_3_-DPA (dihydrophaseic acid), ^2^H_4_-7OH-ABA, ^2^H_5_-ABA-GE (ABA-glucosyl ester) (NRC-PBI), ^2^H_6_-ABA, ^2^H_5_-JA, ^2^H_5_-transZ, ^2^H_5_-transZR, ^2^H_5_-transZ7G, ^2^H_5_-transZ9G, ^2^H_5_-transZOG, ^2^H_5_-transZROG, ^2^H_5_-transZRMP, ^2^H_3_-DHZ, ^2^H_3_-DHZR, ^2^H_3_-DZRMP, ^2^H_7_-DZOG, ^2^H_3_-DHZ9G, ^2^H_7_-DZOG, ^2^H_6_-iP, ^2^H_6_-iPR, ^2^H_6_-iP7G, ^2^H_6_-iP9G, ^2^H_6_-iPRMP (Olchemim). Extracts were purified using a mixed mode reverse phase–cation exchange SPE column (Oasis-MCX, Waters). Two hormone fractions were sequentially eluted: (1) fraction A, eluted with methanol containing ABA, IAA, SA, and JA, and (2) fraction B, eluted with 0.35 M NH_4_OH in 60% methanol containing CKs. Hormone metabolites were analyzed using HPLC (Ultimate 3000, Dionex) coupled to a hybrid triple quadrupole/linear ion trap mass spectrometer (3200 Q TRAP, Applied Biosystems). Quantification of hormones was done using the isotope dilution method with multilevel calibration curves (R2 > 0.99). Data processing was carried out with Analyst 1.5 software (Applied Biosystems). Data are presented as means ± standard error. Three biological replicates were analyzed.

### 2.5. Gene Expression (qPCR)

In every experimental variant, 50 mg (FW) leaf samples were collected on the first and the last day of the treatments. Total RNA was isolated with Direct-zolTM RNA MiniPrepkit (Zymo Research Corp., Irvine, CA, USA) and its quantity was determined by NanoDrop 2000 spectrophotometer (Thermo Fisher Scientific Inc., Wilmington, MA, USA). According to the manufacturer’s protocol, cDNA libraries were prepared with the Moloney Murine Leukemia Virus (M-MLV) Reverse Transcriptase and oligo (dT)_18_ primer (Promega Corporation, Madison, WI, USA). For the analysis of the gene expression levels CFX96 TouchTM real-time PCR Detection System (Bio-Rad Hungary Ltd., Budapest, Hungary) and KAPA SYBR^®^ FAST, Master Mix (2×), Universal qPCR Kit (Kapa Biosystems, Inc., Wilmington, MA, USA) were used. With respect to the qPCR primers, some were our own design and some were as previously published (Supplementary Table S1; [10]). Cyclophilin was used as reference gene and relative expression levels were calculated by the ΔΔCt method [73].

### 2.6. Statistical Analysis

For the statistical evaluation of the data, SPSS 16.0 was used. The homogeneity of the variances was checked by Levene’s test, and normality was tested by Kolmogorov–Smirnov probe. To explore differences between treatments, a one-way ANOVA test was performed. Then, in justified cases additional Dunnett T3 or Tukey’s-b post hoc tests were applied. Student’s t test was used to evaluate the results of the freezing test.

## 3. Results

### 3.1. The Effects of FR-Supplemented Light on Freezing Tolerance

**Figure 2:**
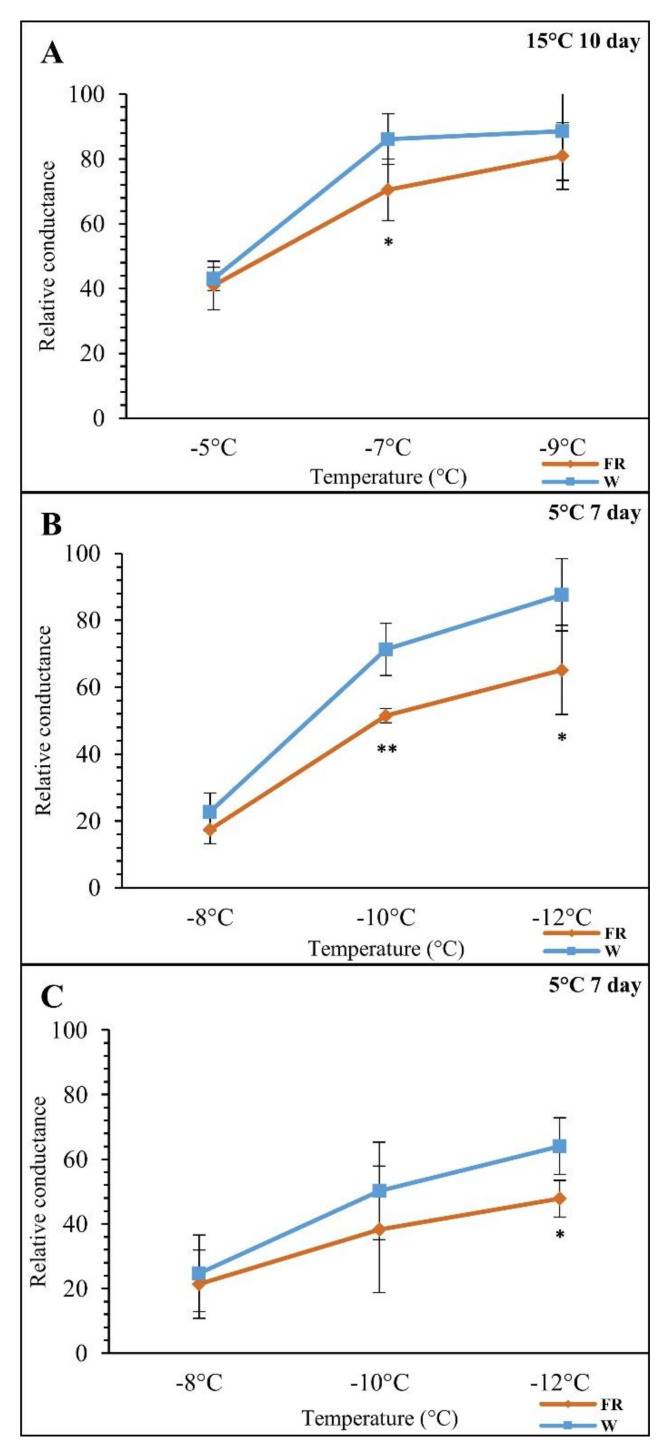
The effects of the supplementary far-red light treatments (FR) on plants’ freezing tolerance determined on the basis of electrolyte leakage. The relative conductance values correspond to percentage of lethality. (**A**) (FR-M): FR treatment at 15 °C for 10 days (treatment began on day 18). (**B**) (FR-M/FR-C(28)): FR pre-treatment at 15 °C for 10 days followed by cold stress at 5 °C for 7 days (cold treatment began on day 28). (**C**) (FR-C(18)): Combined cold (5 °C) and FR treatment for 7 days (treatment began on day 18). W: white light; FR: far-red-enriched light. The data are mean values from five biological replicates. Significance of difference between the control W, and the FR treated samples is designated as * *p* < 0.05 and ** *p* < 0.01 with Student’s *t* test.

The effect of FR on freezing tolerance was evaluated in barley leaves. The comparison of supplementary FR treatment at 15 °C and at 5 °C, both after FR pre-treatment (at 15 °C) and simultaneously with the temperature drop, revealed significant differences among the experimental variants. The electrolyte leakage measurements showed a positive effect of FR supplementation (Figure 2). The results shown in Figure 2A,B were already mentioned in our study of the effect of FR and cold on lipid composition [68]. Here we analysed three treatments (Figure 2C). In the first treatment at 15 °C (FR-M), the FR effect reached statistical significance only at a freezing temperature of −7 °C. The most remarkable freezing tolerance improvement between the W control and the FR-treated samples was observed at prolonged FR exposure FR-M/FR-C(28) (i.e., FR pre-hardening + low temperature treatment at high FR presence), both at -10 °C as well as at −12 °C. In the case of the combination of cold and light treatment (FR-C(18) and the corresponding W control), significant change occurred at a freezing temperature of −12 °C. Membrane damage was lower after plant acclimation to low temperature (5 °C) than in plants grown at 15 °C (Figure 2A) independently from light quality (Figure 2B,C). In the case of the younger seedlings (18 days old at the beginning of the treatment) further improvement was observed both in the control W samples and in the FR supplementation, which further enhanced freezing tolerance (Figure 2C). The results indicate that the FR enrichment positively influences the plant’s freezing tolerance at low temperature. It may be concluded that freezing tolerance is affected by low temperature, FR enrichment in the case of W, as well as plant developmental stage.

### 3.2. Alterations in Plant Hormone Levels during Treatments

**Figure 3:**
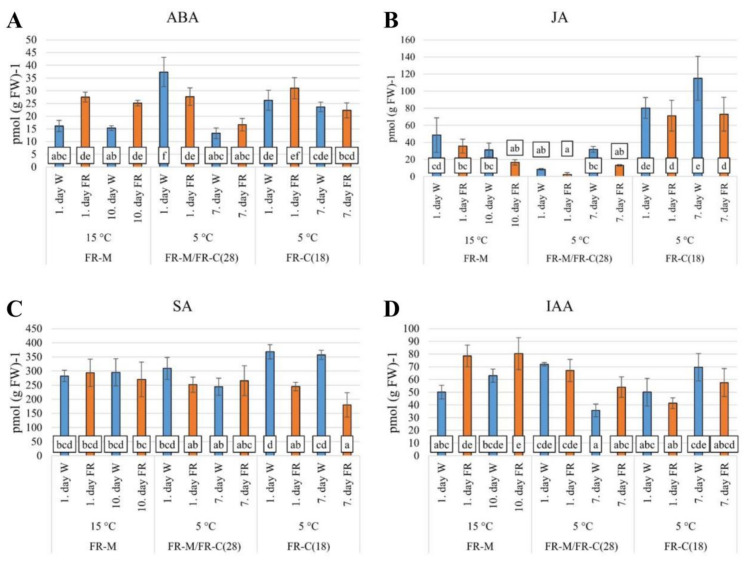
The impact of FR enrichment and moderate or low temperature on hormone concentrations. (**A**): ABA, abscisic acid; (**B**): JA, jasmonic acid; (**C**): SA, salicylic acid; (**D**): IAA, indole-3-acetic acid. Leaf samples were collected six to eight hours after the start of illumination. Plantlets were grown under 12 h photoperiod. FR-M: FR treatment at 15 °C for 10 days. FR-M/FR-C(28): FR treatment at 15 °C for 10 days + FR treatment at 5 °C for 7 days. FR-C(18): FR treatment at 5 °C for 7 days. Plant age at the start of the cold treatment was 28 days in the case of FR-M/FR-C(28) and 18 days in FR-C(18) variant. W: white light, FR: far-red-enriched white light. Mean and standard deviation values were calculated from three biological replicates. Significant differences between the columns were determined by Tukey’s-b test (*p* < 0.05).

**Figure 4:**
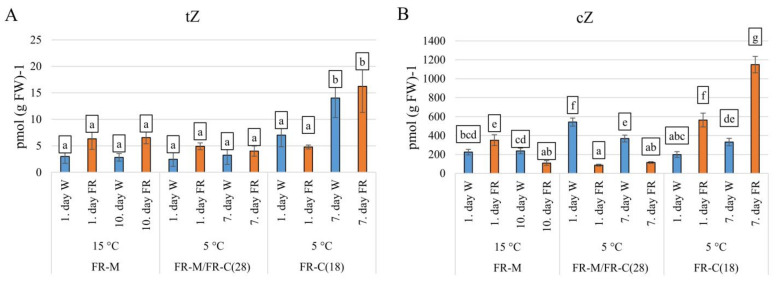
The impact of FR enrichment and moderate or low temperature on active cytokinin concentrations. (**A**): tZ, *trans*-zeatin; (**B**): cZ, *cis*-zeatin. Conditions are the same as in Figure 3.

As phytohormones regulate cold acclimation in an intensive cross-talk, five hormones were followed in the experiments. The impact of FR was compared at different temperatures, treatment lengths, as well as plant ages. Significant differences were detected in the hormone levels and their metabolism (Figure 3 and Figure 4, Supplementary Table S2), namely in case of ABA, JA, SA, IAA, and CKs, and especially of *cis*-zeatin-type.

FR treatment at 15 °C (FR-M) almost doubled the ABA content after 7 h (Figure 3A), and that difference remained steady during the following 10 days. As expected, the cold treatment (5 °C) increased significantly (*p* < 0.05) the ABA content in the control W illuminated samples after 7 h, surpassing the level of the FR-treated samples. The temperature drop had only a moderate effect on the ABA concentration in the FR-acclimated samples after 7 h of cold. After 7 days of cold treatment, ABA content decreased both in the control W- and in the FR-treated samples (Figure 3A, FR-M/FR-C(28)). At the beginning of the FR-C(18) treatment the plantlets were younger (18 days old) than at the FR-M/FR-C(28) (28 days, Figure 1) when the temperature dropped to 5 °C. Another difference was that in the case of FR-C(18) the experimental variant FR-treatment started together with the temperature shift. Both the W control and the FR-treated plants responded similarly to low temperature, although the ABA content in the FR-treated samples surpassed that of the W control samples after 7 h. Most likely the combination of the two external abiotic factors affected ABA metabolism synergistically. Interestingly, the elevated ABA content was partially preserved after a seven-day cold treatment both in the W- and FR-treated samples, a phenomenon that can be explained by the maintenance of an activated defense level in younger plants.

FR supplementation had a moderate negative effect on JA levels at 15 °C (2-fold decrease after 10 days, Figure 3B). The low temperature caused a transient down-regulation of JA content in all the samples. FR pre-treatment strengthened the cold effect in the FR-treated plants (4-fold decrease), especially at the early phase of the response. In the case of cold treatment of young plants (18-d old), JA content was doubled within the first day of the cold stress, irrespective of light treatment, and increased still further after 7 days at 5 °C in W. The FR-treated plants maintained the level reached after 7-h of cold.

Neither FR nor cold treatments affected SA content significantly during the first two treatments (FR-M, FR-M/FR-C(28) and the corresponding W controls, Figure 3C). During cold treatment of young plants (18-d old), SA content changed similarly to JA. Upon cold exposure, SA content moderately increased in W, whereas this elevation was inhibited significantly (*p* < 0.05) by FR-supplementation.

IAA content (Figure 3D) was up-regulated almost two-fold during the first day of FR treatment at 15 °C, and that increase remained steady during the next 10 days (FR-M). This phenomenon is most likely related to the shade avoidance syndrome caused by FR-enriched incident white light. The early response to cold was associated with IAA up-regulation in both the W- and FR-treated plants (FR-M/FR-C(28)). Nevertheless, in the last day of stress a moderate down-regulation was observed in the W illuminated samples. The FR treatment caused partial maintenance of IAA elevation. In the FR-C(18) experimental variant, combined stress caused a transient IAA down-regulation in FR-treated plants. After 7 days, the moderate IAA elevation may have indicated acclimation in the younger plants, irrespective of light treatment.

The levels of active CKs, *trans*-zeatin (tZ), dihydrozeatin (DHZ), and isopentenyladenine (iP) were low in comparison with *cis*-zeatin (cZ). The most physiologically active CK, *trans*-zeatin (tZ) was moderately enhanced by FR at 15 °C (FR-M). After 10 days, no significant change was detected (Figure 4A). Cold stress resulted in transient tZ decrease in white light. Plants pre-treated with FR (FR-M/FR-C(28)) were able to maintain higher tZ levels at low temperature. At the end of the stress treatment, slight tZ up-regulation was observed in the comparison with W exposed plants. Combination of FR and cold (FR-C(18)) had no significant effect during the first day; however, significant elevation was found after 7 days of cold, irrespective of light treatment.

FR-treatment caused transient up-regulation in cZ content at 15 °C (FR-M) followed by its down-regulation (Figure 4B). After the temperature decrease to 5 °C (FR-M/FR-C(28)), cZ content increased significantly in the W illuminated samples. After FR pre-treatment, the supplementary FR light repressed this cold-induced elevation. In contrast, simultaneous application of cold and FR (FR-C(18)) enhanced cZ content 3-fold during 1 day compared to the W illuminated samples. The cZ level was further enhanced after 7 days.

### 3.3. The Expression Patterns of the Key Hormone Metabolism-Related Genes

**Figure 5:**
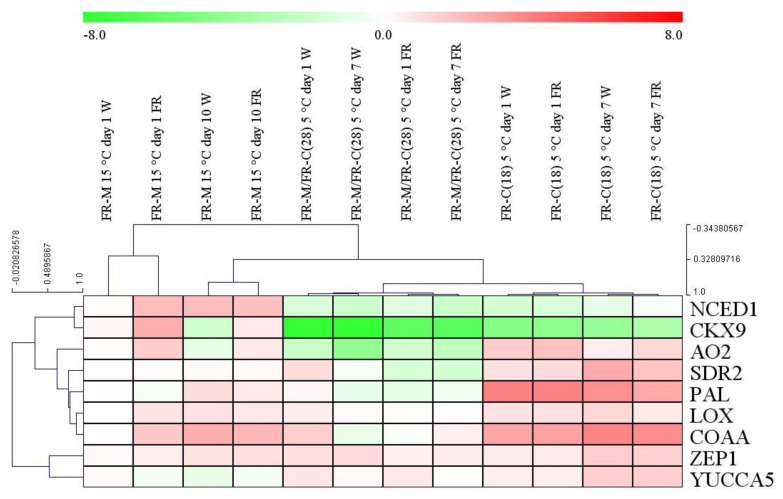
The effect of supplementary FR light and low temperature on the relative expression of the genes related to the hormone metabolism. Leaf samples were collected six to eight hours after the start of illumination. The plantlets were grown under 12 h photoperiod. Transcript levels were calculated with the ΔΔCt method. Log_2_ expression values are shown. W: white light, FR: far-red-enriched white light. The data originated from three biological replicates. The values on the X and Y axis outside of the heatmap refer to the distance or proximity of data after hierarchical clustering.

The expression of selected hormone metabolism-related genes was determined by qPCR. All the relative expression values were compared to the first day of the first treatment at 15 °C (FR-M). The results are shown in the heatmap (Figure 5).

The treatments clustered separately based on the transcript levels, which clearly showed the importance of the timing of the FR treatments.

Transcription profiles of the ABA biosynthetic genes *ZEP1*, *NCED1*, *SDR2,* and *AO2* were determined. The expression of *ZEP1* varied only slightly during FR-M and FR-M/FR-C(28) treatments. In the FR-C (18) experiment, when the younger plants were cold treated, *ZEP1* transcript levels were elevated after 7 days independently from light quality. A large positive effect of FR light on *NCED1* expression was detected at 15 °C. At low temperature, *NCED1* expression was strongly down-regulated, independently from plant age or light spectra. The other two genes (*SDR2* and *AO2*) behaved similarly, and were clustered very closely together. Their expression was down-regulated, mainly by low temperature, except during the first day of the FR-M experiment, where the expression of *AO2* was slightly increased. The supplementary FR light, in synergy with the cold, further reduced transcript levels. In the FR-C(18) treatment this phenomenon was quite the opposite: instead of the decreasing pattern, an elevation was observed.

The genes related to JA and SA metabolism (*LOX* and *PAL)* belong to the same cluster, together with some ABA-related genes. The FR-treatment caused a mild decrease in the expression of these two genes after 10 days at 15 °C. An opposite pattern was observed in the FR-M/FR-C(28) and FR-C(18) experiments, the former one was associated with a mild down-regulation, whereas the latter one with up-regulation (especially of *PAL*). The cold treatment of 18-day-old plants induced their expression independently from light quality in the third treatment.

**Figure 6:**
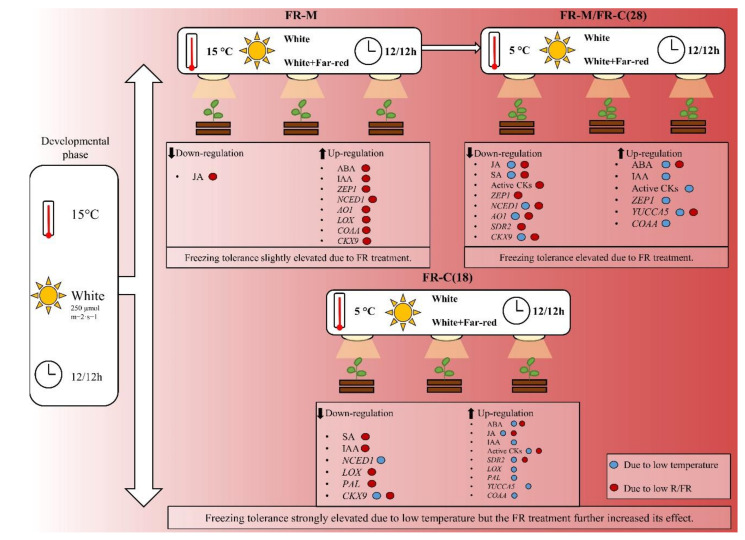
Summary of phytohormone responses and changes in the expression profiles of the key hormone metabolism-related genes elicited by low temperature treatment and/or FR enrichment. FR-M (Treatment 1): The plantlets were exposed to high FR at the age of 18–28 days under constant 15 °C. FR-M/FR-C(28) (Treatment 2): The plantlets were subjected to high FR for 10 days at 15 °C, then the temperature was dropped to 5 °C for seven days (at high FR). FR-C(18) (Treatment 3): The plantlets were 18 days old when the temperature was dropped to 5 °C for seven days (at high FR). ABA: abscisic acid; IAA: indole-3-acetic acid; JA: jasmonic acid; SA: salicylic acid; cZ: *cis*-zeatin; active CKs: active cytokinins; CKX9: cytokinin oxidase/dehydrogenase 9; COAA: acetyl-CoA acetyltransferase; YUCCA5: indole-3-pyruvate monooxygenase; ZEP1: zeaxanthin epoxidase; NCED1: 9-cis-epoxycarotenoid dioxygenase1; SDR2: short-chain dehydrogenase; AO2: aldehyde oxidase 2; LOX: lipoxygenase; PAL: phenylalanine ammonia-lyase.

The *COAA* and *YUCCA5* genes were investigated as important genes in auxin biosynthesis. The largest changes were observed in the case of *COAA*. A 3-fold stimulation of expression was caused by FR supplementation at 15 °C after 7 h (FR-M). Comparison of FR-M/FR-C(28) and the corresponding W control showed that low temperature eliminated the differences between light treatments. The cold treatment of 18-day-old plants resulted in even larger increases, independently from light quality. The expression pattern of *CKX9* was clustered to *NCED1.* A large, six-fold elevation of *CKX9* expression was observed upon FR supplementation to W at 15 °C (FR-M). At low temperature, *CKX9* expression decreased dramatically (Figure 6-FR-M/FR-C(28) and FR-C(18) treatments).

In general, except for *CKX9*, the larger changes occurred mostly upon the cold exposure of 18-day-old plants. In this experimental setup the treated plants were not pre-acclimated to FR enriched light, and were still at the beginning of the three-leaf stage. Similarly to phytohormone content, the most pronounced interaction between FR and cold treatments was displayed here.

## 4. Discussion

### 4.1. FR Impact at Moderate Temperature

Plants perceive light by different photoreceptors. The only photoreceptor activated by FR is phytochrome A (PhyA). This photoreceptor was reported to enhance plant freezing tolerance [67,74,75]. The positive effect of the enhanced FR/R ratio associated with PhyA activation is in accordance with our data on FR-induced promotion of freezing tolerance in barley at 15 °C (Figure 2). This finding is in line with our previous study [61], where a positive effect of FR enrichment on the expression of the key cold-inducible transcription factor *CBF14* was demonstrated at 15 °C. However, the precise mechanism of the FR effect on cold acclimation has not yet been revealed. In our previous studies we demonstrated in cereals (both in wheat and barley) that an effect of FR can be further increased by higher light intensity [61,62]. It should also be mentioned that in spring barley varieties grown at normal growth temperature, FR light is less effective than other modifications like blue light enrichment in the illuminating light [76].

Apart from the effect of FR on freezing tolerance, an adaptation reaction to autumnal changes in the composition of the light spectrum, the enhanced FR/R ratio may also be sensed by plants as a marker of shading, because plants use R light for photosynthesis and reflect FR. In dense vegetation this provokes the shade avoidance syndrome, which in dicots is associated with promotion of hypocotyl/stem growth and suppression of leaf development [77], and in monocots with stem elongation and subsequent reduced number of spikelets and filled grains [78]. These developmental changes, as well as cold acclimation, are regulated by plant hormones, and thus their change imposed by FR exposure might be related to cold hardening, shade avoidance syndrome, or both.

The most prominent change after FR supplementation at 15 °C was the up-regulation of ABA content (Figure 3A), together with stimulation of the expression of the gene for its rate-limiting biosynthetic enzyme NCED1, as well as of other ABA biosynthetic genes such as *AO2*, and, to a lesser extent, *ZEP1* and *SDR2* (Figure 5). This is in accordance with other studies, which reported that genes related to ABA biosynthesis are strongly up-regulated by FR supplementation [79,80]. The low R/FR ratio can up-regulate ABA in *A. thaliana* in order to inhibit bud outgrowth [81]. The function of ABA in cold hardening seems to be given (at least partially) by stimulation of the expression of protective substances, e.g., LEA proteins [5], or by suppression of growth [82].

Down-regulation of JA during prolonged FR exposure at 15 °C (Figure 3B) is in accordance with its suppression during shade response, associated with enhanced plant vulnerability to herbivore attack at low R/FR ratio [83]. In *A. thaliana,* low R/FR ratio repressed the JA-induced defense responses, probably due to the fact that plants need to relocate their energy resources from the stimulation of defense to rapid elongation [84]. FR light also negatively influenced JA biosynthesis in spring barley leaves [76]. Apart from diminished JA content, its signalling pathway was also reported to be suppressed, as FR-induced phytochrome B inactivation leads to stabilization of JA repressors–JAZ proteins (JASMONATE ZIM DOMAIN) [85].

The low R/FR ratio adversely affects not only JA-dependent but also SA-dependent defenses in *A. thaliana* [86]. The shade-avoidance response is prioritised over plant immune responses [84,86]. However, only mild SA decrease was detected in barley upon prolonged FR (Figure 3C).

The hormone generally elevated during the shade avoidance response is IAA [87,88]. Indeed, significant up-regulation of this auxin was detected during the whole period of FR exposure at 15 °C (Figure 3D), together with promotion of the expression of *COAA* (Figure 5). It is known that IAA over-producing plants have an up-regulated activity of CKX, the main CK-degrading enzyme [77,89]. The increase in IAA coincided with significant up-regulation of *CKX9* (Figure 5). This correlates well with the decrease of active CKs after 10 days at high FR (Figure 4), and is in accordance with the fact that shade avoidance is usually associated with a decrease in CKs, related to inhibition of leaf growth (in favour of hypocotyl elongation) [90].

### 4.2. The Effect of FR Pre-Treatment on Cold Stress Response

The results of the electrolyte leakage analysis (Figure 2) showed that pre-treatment with FR had a strong positive effect on freezing tolerance after subsequent cold treatment at 5 °C (FR-M/FR-C-(28)). Decrease of the temperature to 5 °C resulted, in W grown plants, in a strong, transient up-regulation of ABA (Figure 3A). This rise of ABA is in accordance with previous reports [5,91]. Thus, low temperature exhibited a similar effect on ABA metabolism as FR treatment at 15 °C. Cold, among other stress cues (salinity, dehydration, heat), strongly influences ABA levels by activating many genes involved in the biosynthetic pathway [92]. Plant priming with FR diminished the subsequent cold-induced ABA elevation during one day of cold stress. The reason for this may be an enhanced cold tolerance caused by FR pre-treatment, which diminished the stress strength, and was thus associated with lower stress response. Later on, the level of ABA decreased in both light regimes. The ABA decrease during prolonged cold treatment is in accordance with previous reports, e.g., [5]. The ABA profile coincided with the expression of *SDR2* and *AO2*. Low *NCED1* transcript level may indicate that ABA elevation in leaves was promoted by its transport from the roots.

JA levels were rather low at the early cold stress response (Figure 3B) and moderately elevated later on. This is in accordance with the report by [93]. The negative effect of FR, observed already at 15 °C, was maintained during the whole cold period (Figure 3B). Only minor changes were observed in the case of SA (Figure 3C). Moderate down-regulation of the expression of *PAL* agrees with a mild decrease in SA under FR at the beginning of the cold period.

The effect of FR on IAA was abolished during one day at 5 °C (Figure 3D), which indicates that temperature has a stronger impact than light composition. Later on, the positive effect of FR was clearly visible. Cold stress had a moderate negative effect on tZ, the most active CK in stimulation of cell division. In contrast, content of cZ, the low active CK often related to stress responses, increased. Later on, tZ slightly increased, whereas cZ moderately decreased, which may indicate plant acclimation to low temperature. In accordance with the treatment at 15 °C, FR also exhibited a negative effect on cZ at 5 °C. The slightly higher tZ content in FR-treated plants during 1 day may reflect higher stress tolerance given by FR pre-treatment and thus a diminished stress effect.

### 4.3. Combined FR and Cold Stress

In the FR-C(18) treatment, cold stress was applied simultaneously with FR supplementation to young 18-day-old plants. In comparison with the FR-M/FR-C(28) experiment, younger plants already exhibited significantly higher stress tolerance in white light (Figure 2). It seems that plants are more sensitive to low temperature during the three leaf developmental stage compared to later stages of their development. The early elevation of ABA was slightly lower in comparison with 28-day-old plants. The most plausible explanation for this phenomenon is the following: In the temperate region of the northern hemisphere the sowing period of winter cereals begins in the first–second week of October and continues until November. Consequently, when the first frosty spells affect the developing plants (night frost generally appears at the beginning of November) they are still in the very young phase of their development (2–3 leaf stage). So, for several hundred years the farmers/breeders selected unintentionally for variants which were able to adapt to this harsh climate. Indeed, it was published recently that light quality and quantity affected wheat and barley growth and development and even the output of circadian clock [65,94,95,96]. Simultaneously, up-regulation of JA and SA was much more profound than in the FR-M or FR-M/FR-C(28) experiments. The hormone profiles are in accordance with the expression patterns of their biosynthetic genes; strong stimulation was detected in the case of SA-related gene *PAL* and, to lesser extent, of JA-related gene *LOX*. This is in accordance with previous reports, which showed that low temperature or cold acclimation usually results in elevated PAL activity, e.g., in festulolium or rape plants [97,98]. Most likely, the plantlets prioritize keeping their defense mechanisms alert against extreme environmental conditions in this developmental stage. At the end of the 7-day cold period, all stress hormones, especially JA, were elevated. Stimulation of LOX was in contrast to the results of Liu et al. [99], who reported cold-induced suppression of several *LOX* genes in oriental melon. However, the response may be species specific and, moreover, various LOX genes may be subject to different modes of regulation.

The combination of cold and FR was found to induce an enhanced cold acclimation, compared to cold alone, since the level of ABA was increased, while IAA was decreased. The difference in ABA accumulation between FR-M/FR-C(28) and FR-C(18) may have been due to FR pre-treatment in the former case, which is in accordance with the difference between non-acclimated and cold-acclimated barley [100]. FR caused a substantial increase of the stress-related CKs (cZ). FR imposed a significant negative effect on JA after 7 days (as during shade response), strongly suppressing SA levels. Both hormone levels and the expression profiles of hormone metabolism-related genes indicated that an activated defense was maintained during the whole cold treatment. The increase in IAA (together with high CK content), which is in accordance with up-regulation of *YUCCA5* and *COAA* expression, seems to indicate that young plants were able to acclimate better to stress conditions.

### 4.4. Summary

FR-supplemented white light acts as a coordinator in the pre-hardening process, aside from its regulation of many plant developmental and physiological processes. Supplementary FR light had a positive impact on the plants’ cold tolerance, even at moderate temperature. This effect was strengthened by cold treatment at 5 °C, in comparison with a non-cold hardening temperature. The results of the hormone and the qPCR analyses indicate that the combination of these two environmental factors enhanced some impacts, as well as suppressing the other imposed by FR or cold separately. A summary of the data is shown in Figure 6. The synergistic effects of these two environmental cues on freezing tolerance indicate that their combination represents a unique environmental signal, which is a part of plant adaptation to seasonal variations in temperate zones. Even if the effect of low temperature had a greater impact on plant behaviour than modification of light spectrum, the timing and duration of the FR treatment seem to be crucial factors in cold acclimation processes, as far as plant acclimation is concerned. Another important factor is plant age. Cold hardening was more efficient in the early developmental stages of barley plants, which was associated with higher stimulation of plant defenses, as indicated by the comparable elevation of ABA and higher increase of JA and SA.

## 5. Conclusions

Characterization of the FR effects on dependence on temperature showed a close relationship between plant hormones, light quality, and low temperature during the cold acclimation process. The significant modulation of phytohormone pools and the related transcriptome by the FR enrichment at moderate or low temperature was in all cases associated with elevated frost tolerance. Comparing the response of plants at the three-leaf stage and at later developmental stage revealed that the concentrations of SA, JA, and the active CKs exhibited significant promotion in the younger plants. As the experimental system presented here mimicked well the late autumn situation in the temperate and boreal climates of the northern hemisphere, it seems that elevated protection against low temperature can occur in plants as an adaptation to autumn-related environmental changes, distinct from the shade avoidance syndrome.

## Supplementary Materials

The following are available online at https://www.mdpi.com/2218-273X/11/3/450/s1.
