# Supplementary material for: The Impact of Far-Red Light Supplementation on Hormonal Responses to Cold Acclimation in Barley
Source: Biomolecules. 2021 Mar 17;11(3):450. doi: 10.3390/biom11030450 (PMC8002655; doi:10.3390/biom11030450)
Supplement: Supplementary file 1 [file biomolecules-11-00450-s001.pdf]

biomolecules

Supplementary Materials

The Impact of Far-Red Light Supplementation on Hormonal Responses to Cold Acclimation in Barley

Mohamed Ahres, Tamás Pálmai, Krisztián Gierczik, Petre Dobrev, Radomíra Vanková and Gábor Galiba

[1] Centre for Agricultural Research, Agricultural Institute, Eötvös Loránd Research Network, H-2462 Martonvásár, Hungary; mohamed.ahres@atk.hu (M.A.); palmai.tamas@atk.hu (T.P.); gierczik@gmail.com (K.G.); galiba.gabor@atk.hu (G.G.)
[2] Department of Environmental Sustainability, Festetics Doctoral School, IES, Hungarian University of Agriculture and Life Sciences, H-8360 Keszthely, Hungary
[3] Institute of Experimental Botany of the Czech Academy of Sciences, 165 02 Prague, Czech Republic; dobrev@ueb.cas.cz
* Correspondence: vankova@ueb.cas.cz

Citation: Ahres, M.; Pálmai, T.; Gierczik, K.; Dobrev, P.; Vanková, R.; Galiba, G. The impact of far-red light supplementation on hormonal responses to cold acclimation in barley. **2021**, *11*, x. https://doi.org/10.3390/xxxxx

Academic Editor: Guzel Kudoyarova

Received: 11 January 2021
Accepted: 16 March 2021
Published: date

Publisher's Note: MDPI stays neutral with regard to jurisdictional claims in published maps and institutional affiliations.

Copyright: © 2021 by the authors. Submitted for possible open access publication under the terms and conditions of the Creative Commons Attribution (CC BY) license (http://creativecommons.org/licenses/by/4.0/).

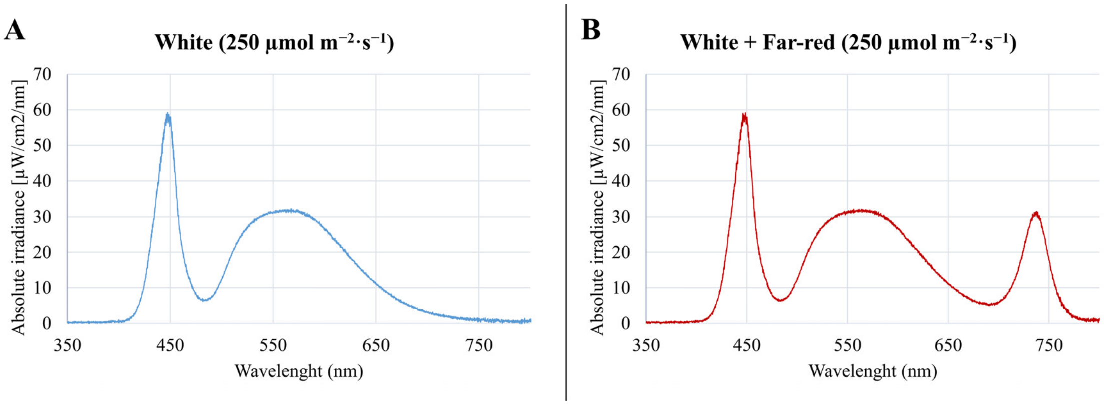

Figure S1. Spectral composition of modulated light treatments at 250 µmol $m^{-2} \cdot s^{-1}$ intensity. **A)** the spectral composition of the white light, **B)** the spectral composition of white light supplemented by extra far-red light (Kovács et al., 2020).

Kovács, T., Ahres, M., Pálmai, T., Kovács, L., Uemura, M., Crosatti, C., Galiba, G., 2020. Decreased r:Fr ratio in incident white light affects the composition of barley leaf lipidome and freezing tolerance in a temperature-dependent manner. Int. J. Mol. Sci. 21, 1–24. https://doi.org/10.3390/ijms21207557

Table S1. Primers used for the qRT-PCR.

Name	Abbreviation	Forward	Reverse	Reference
9-cis-epoxycarotenoid dioxygenase	NCED1	CTCTCCTACATCTCGCCTGC	CCTTCTGTAGCTGGGGTTCG	own design
cytokinin dehydrogenase 9	CKX9	TGATCTCTTCCGTGCTGCTC	CGATCCTGGCCCGAGTAATG	own design
aldehyde oxidase 2	AO2	CGCTCTCTCGTTCGACATCA	GGAGCCCGCATTACTGACTT	own design
short-chain dehydrogenase 2	SDR2	GCCATCTCGCCCAACTACATCC	CGGCGCCCTCCATCTCGT	Seiler et al., 2011
phenylalanine ammonia-lyase	PAL	TGCTCGTCCGTGTCAATACC	CGTTGGCGTTGAGAAGTGTG	own design
lipoxygenase	LOX	GAAGGTGGAGGTCAAGGAGC	CCCCGACTTGTTGCTCTTCT	own design
acetyl-CoA acetyltransferase	CoAA	ATTGTTGTGGCAGGTGGGAT	CAACGAGGCTGTCATGTCC	own design
zeaxanthin epoxidase	ZEP1	GCGAGAGGCGGGGGAGAAGT	TGGTGACAAGGGGTGGCTGAAG	Seiler et al., 2011
indole-3-pyruvate monooxygenase	YUCCA5	GCAGCAGACAACAACAGCAA	CTTTTGCTTTGGTGGCTGCT	own design

Seiler, C., Harshavardhan, V.T., Rajesh, K., Reddy, P.S., Strickert, M., Rolletschek, H., Scholz, U., Wobus, U., Sreenivasulu, N., 2011. ABA biosynthesis and degradation contributing to ABA homeostasis during barley seed development under control and terminal drought-stress conditions. J. Exp. Bot. 62, 2615–2632. https://doi.org/10.1093/jxb/erq446

Table S2. Table summarizing the results of the hormone analysis FR-M: FR treatment at 15°C for 10 days. FR-M/FR-C(28): FR pre-treatment at 15°C for 10 days followed by cold stress at 5°C for 7 days (cold treatment began on day 28). FR-C(18): Combined cold (5°C) and FR treatment for 7 days (treatment began on day 18). W – white light; FR - far red-enriched light.

Treatments	Temperatures (°C)	Time/Light Regimes	Avarages/Standard Deviations	ABA	PA	9OH-ABA	IAA	OxIAA	PAA	SA	JA	cis-OPDA	cZ	tZ	iPR	cZR	cZOG	iP7G	iP9G
**FR-M**	15°C	day 1 W	AVG.	16.15	36.61	15.93	50.11	35.04	759.37	282.60	48.55	5164.41	224.42	3.01	17.51	246.67	68.35	28.65	0.60
			SD	2.18	6.66	3.15	5.35	2.72	98.89	19.96	20.21	1943.06	29.43	1.31	5.77	10.15	3.58	5.72	0.67
		day 1 FR	AVG.	27.49	75.05	15.48	78.55	40.58	999.72	293.83	35.61	1335.20	350.48	6.32	10.62	265.34	68.55	29.59	2.23
			SD	1.94	20.07	4.87	8.53	5.78	34.90	48.17	8.10	122.38	57.17	1.99	0.90	32.83	8.93	1.02	3.13
		day 10 W	AVG.	15.30	114.28	30.01	63.04	38.74	686.19	295.61	31.06	3238.08	237.18	2.82	12.91	180.22	117.40	34.94	1.61
			SD	0.98	13.51	2.19	5.13	3.21	37.14	48.05	7.98	669.72	29.59	0.71	0.78	33.44	8.82	0.93	0.57
		day 10 FR	AVG.	25.15	96.09	24.34	80.34	43.36	826.44	270.33	16.48	466.24	109.06	6.57	2.98	138.14	97.66	130.26	1.35
			SD	1.12	11.11	4.32	12.58	9.53	131.37	61.11	3.08	27.57	24.99	1.19	1.91	23.79	5.03	30.91	1.28
**FR-M / FR-C(28)**	5°C	day 1 W	AVG.	37.34	147.39	24.08	71.98	65.41	782.92	309.28	8.44	1287.76	542.16	2.45	14.32	199.74	126.23	44.11	0.47
			SD	5.75	9.28	3.76	1.35	4.84	7.96	38.98	0.97	208.24	42.65	1.31	0.64	34.06	9.82	0.76	0.42
		day 1 FR	AVG.	27.64	93.67	19.19	67.12	33.00	865.42	251.68	2.44	477.63	85.83	4.91	5.40	166.86	86.11	55.02	0.49
			SD	3.46	11.57	0.46	8.76	4.16	86.45	26.81	2.17	47.79	10.10	0.63	1.70	33.03	18.06	12.70	0.47
		day 7 W	AVG.	13.29	183.10	48.42	35.67	75.34	355.05	244.47	31.71	511.88	368.06	3.22	3.96	94.61	106.06	55.01	4.48
			SD	2.08	13.19	4.18	5.04	10.05	13.14	30.23	3.45	25.15	35.28	1.71	1.67	9.18	10.54	6.38	3.85
		day 7 FR	AVG.	16.64	114.49	17.15	53.96	29.85	480.22	265.86	13.04	447.72	114.57	4.00	4.44	100.54	100.75	23.51	0.25
			SD	2.46	3.13	0.34	8.04	0.09	13.56	53.11	1.01	1.21	9.05	0.96	2.02	21.30	12.30	8.37	0.42
**FR-C(18)**	5°C	day 1 W	AVG.	26.21	183.26	43.74	50.07	43.16	1416.47	368.24	80.32	481.46	198.67	7.01	37.30	91.81	67.03	9.54	0.85
			SD	3.93	17.52	14.90	10.81	5.92	418.86	25.35	12.18	70.20	31.21	2.18	11.40	16.93	9.95	1.08	0.56
		day 1 FR	AVG.	30.99	271.55	46.81	41.34	43.86	3228.98	244.99	71.15	336.91	563.48	4.78	113.62	295.34	68.52	10.50	0.87
			SD	4.16	19.63	10.14	4.24	14.57	337.19	14.76	18.03	39.62	73.72	0.38	16.81	50.64	6.29	2.26	0.46
		day 7 W	AVG.	23.63	169.10	48.12	69.67	40.23	2020.28	357.24	115.09	391.58	330.49	14.03	96.93	185.79	65.42	17.74	1.59
			SD	1.90	23.31	12.92	10.70	13.00	125.26	16.28	25.76	60.64	38.75	3.70	31.33	60.43	4.45	7.53	0.54
		day 7 FR	AVG.	22.26	151.72	40.00	57.57	66.87	2305.86	180.20	73.01	381.76	1149.73	16.24	193.77	343.82	53.63	18.13	0.51
			SD	2.95	41.01	8.42	11.01	15.98	522.77	43.20	19.85	163.96	86.62	4.94	29.67	39.21	7.88	1.27	0.36
